# Solid pseudopapillary neoplasm of the pancreas showing marked distal atrophy: A case report

**DOI:** 10.1016/j.ijscr.2019.01.030

**Published:** 2019-01-30

**Authors:** Masanori Tsujie, Tomoko Wakasa, Shigeto Mizuno, Hajime Ishikawa, Hironobu Manabe, Taichi Koyama, Kotaro Kitani, Shumpei Satoi, Keisuke Inoue, Shuichi Fukuda, Toshihiko Kawasaki, Masao Yukawa, Yoshio Ohta, Masatoshi Inoue

**Affiliations:** a Department of Surgery, Kindai University Nara Hospital, Otoda-cho 1248-1, Ikoma 630-0293, Nara, Japan; b Department of Pathology, Kindai University Nara Hospital, Otoda-cho 1248-1, Ikoma 630-0293, Nara, Japan; c Department of Endoscopic Diagnosis and Treatment, Kindai University Nara Hospital, Otoda-cho 1248-1, Ikoma 630-0293, Nara, Japan; d Department of Gastroenterology, Kindai University Nara Hospital, Otoda-cho 1248-1, Ikoma 630-0293, Nara, Japan

**Keywords:** CT, computed tomography, EUS, endoscopic ultrasonography, EUS-FNA, endoscopic ultrasound-guided fine needle aspiration, ISGPF, international study group of pancreatic fistula, MPD, main pancreatic duct, MRI, magnetic resonance imaging, SPN, solid pseudopapillary neoplasm, Solid pseudopapillary neoplasm, Parenchymal atrophy, Exocrine dysfunction, Central pancreatectomy, Case report

## Abstract

**Introduction:**

Solid pseudopapillary neoplasm (SPN) of the pancreas is a rare neoplasm, affecting primarily young females. Because SPNs are of low-malignancy, they rarely obstruct the main pancreatic duct (MPD) and cause atrophy of the distal pancreas even if their tumor sizes are large.

**Presentation of case:**

A 35-year-old female was referred to our hospital due to pancreatic tumor. Imaging findings showed the presence of well-defined round tumor in the body of the pancreas with 25-mm in diameter. The pancreas parenchyma distal to the tumor was markedly atrophic, and MPD dilatation was not observed. The lesion was diagnosed as SPN by endoscopic ultrasound-guided fine needle aspiration (EUS-FNA), and central pancreatectomy was performed. Intraoperative frozen section of the distal atrophic pancreas showed no evidence of acinar cells, indicating exocrine dysfunction. Therefore, we closed distal pancreas stump instead of reconstruction. In the distal atrophic parenchyma, scattered foci of islets of Langerhans and the vestige of dilated MPD were observed. She has shown neither endocrine nor exocrine insufficiency after surgery.

**Discussion:**

SPNs are usually found without atrophic change of distal pancreas. To the best of our knowledge, this is the first report of SPN in which exocrine dysfunction of atrophic pancreas was demonstrated pathologically and central pancreatectomy without anastomosis of distal pancreas was chosen for the surgical treatment.

**Conclusion:**

We reported a very rare case of SPN with marked distal parenchymal atrophy. We successfully performed central pancreatectomy without reconstruction.

## Highlights

• Solid pseudopapillary neoplasm with marked parenchymal atrophy of the distal pancreas.
• No acinar cells were observed, indicating exocrine dysfunction of atrophic parenchyma.
• The vestige of main pancreatic duct was observed in the distal atrophic pancreas.
• Central pancreatectomy without anastomosis of distal side of pancreas was performed.

## 1 Introduction

The solid pseudopapillary neoplasm (SPN), primarily affecting young women, is a rare epithelial neoplasm, accounting for 1–2% of pancreatic tumors and about 5% of cystic pancreatic tumors [1,2]. SPNs are known as low-grade malignant potential and surgical resection alone offers the patient an excellent chance for long-term survival [[2], [3], [4], [5]]. In addition, less aggressive surgery has been reported to achieve a favorable curative effect for SPNs [6]. Therefore, preoperative histological diagnosis is very important and endoscopic ultrasound-guided fine needle aspiration (EUS-FNA) has become one of the useful diagnostic tools [7]. Compared with other malignant pancreatic tumors, SPNs rarely obstruct the main pancreatic duct (MPD) and cause atrophy of the distal pancreas even if their tumor sizes are large [8]. Here, we report a rare case of SPN in the body of the pancreas causing marked atrophy and exocrine dysfunction of distal pancreas. We successfully performed central pancreatectomy without reconstruction of atrophic distal pancreas. The work has been reported in line with the SCARE criteria [9].

## 2 Presentation of case

A 35-year-old female patient, with no past medical or surgical history, was referred to our hospital due to pancreas tumor, which was identified by abdominal ultrasonography in the medical checkup.

**Fig. 1:**
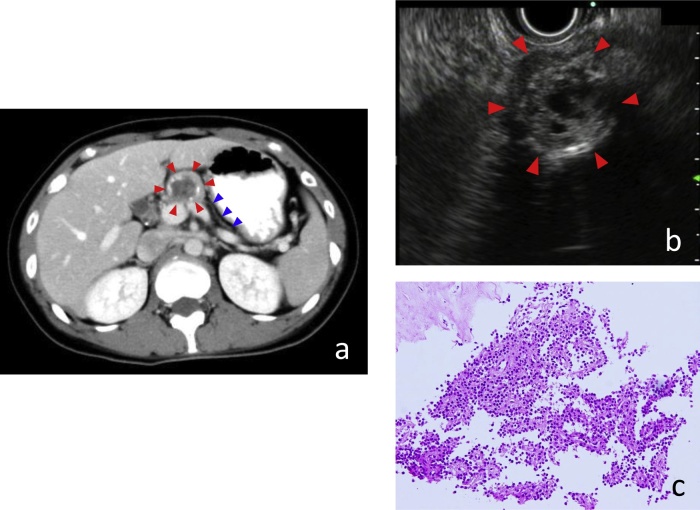
**(a)** Abdominal computed tomography (CT) reveals a 25-mm-diameter round encapsulated mass with peripheral calcification in the body of pancreas (red arrow heads). The solid part shows gradual slight enhancement. The pancreas distal to the tumor is markedly atrophic (blue arrow heads). The dilatation of main pancreatic duct (MPD) is not observed. **(b)** Endoscopic ultrasonography (EUS) shows a 23-mm mass with several cystic components in the pancreas body (red arrow heads). **(c)** Histopathological images of samples from endoscopic ultrasound-guided fine-needle aspiration (EUS-FNA) shows a highly cellular mass with papillary clusters of small and uniform cells with oval nuclei surrounding a fibrovascular core (hematoxylin and eosin stain; original maginification, x200).

An abdominal computed tomography (CT) scan revealed the presence of well-defined round tumor in the body of the pancreas with 25-mm in diameter. The tumor showed good encapsulation, solid and cystic component, and peripheral calcification, and the solid part showed gradual slight enhancement. The pancreas distal to the tumor was markedly atrophic (Fig. 1a). Dilatation of main pancreatic duct (MPD) was not observed. No ascites or metastases to other organs or lymph nodes was evident. EUS showed a 23-mm heterogenous mass with several small cystic components (Fig. 1b). For histological diagnosis, she subsequently underwent EUS-FNA. The tumor was found to be a highly cellular mass with papillary clusters of small and uniform cells with oval nuclei surrounding a fibrovascular core, confirming the preoperative diagnosis of SPN (Fig. 1c).

**Fig. 2:**
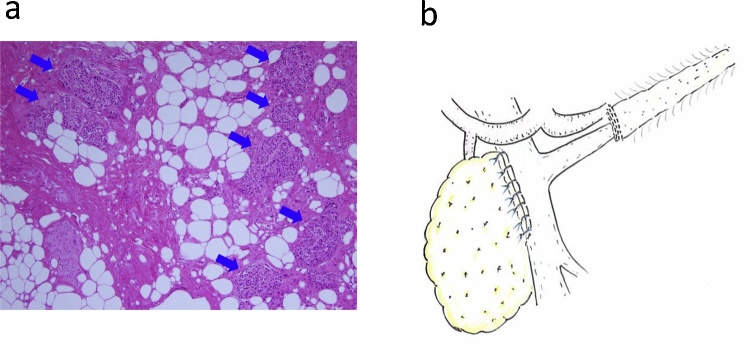
**(a)** Intraoperative frozen section of the distal pancreas margin shows no evidence of acinar cells, which indicates the exocrine dysfunction, although scattered foci of islets of Langerhans are observed (blue arrows), corresponding to end-stage chronic pancreatitis. **(b)** Central pancreatectomy without reconstruction of the distal pancreas was performed.

Based on these results, distal pancreatectomy was planned. First, we dissected pancreas head about 1-cm proximal to the tumor. Pathological diagnosis during surgery showed that no tumor invasion was observed on the proximal margin. Next, we proceeded to separate the tumor and adjacent pancreas from the major retropancreatic vessels toward the distal side. However, it was quite difficult to tunnel under the distal atrophic pancreas because of the inflammatory adhesions. Therefore, we dissected the pancreas about 1-cm distal to the tumor. Intraoperative frozen section of the distal cut-end showed no tumor invasion. Moreover, there was no evidence of acinar cells in the distal atrophic pancreas, which indicated the exocrine dysfunction, although scattered foci of islets of Langerhans were observed (Fig. 2a). As a result, we chose to perform central pancreatectomy and close pancreas distal stump with the automatic suturing device instead of pancreaticojejunostomy or pancreaticogastrostomy (Fig. 2b). The proximal stump was closed by interrupted sutures after the proximal main pancreatic duct was ligated.

**Fig. 3:**
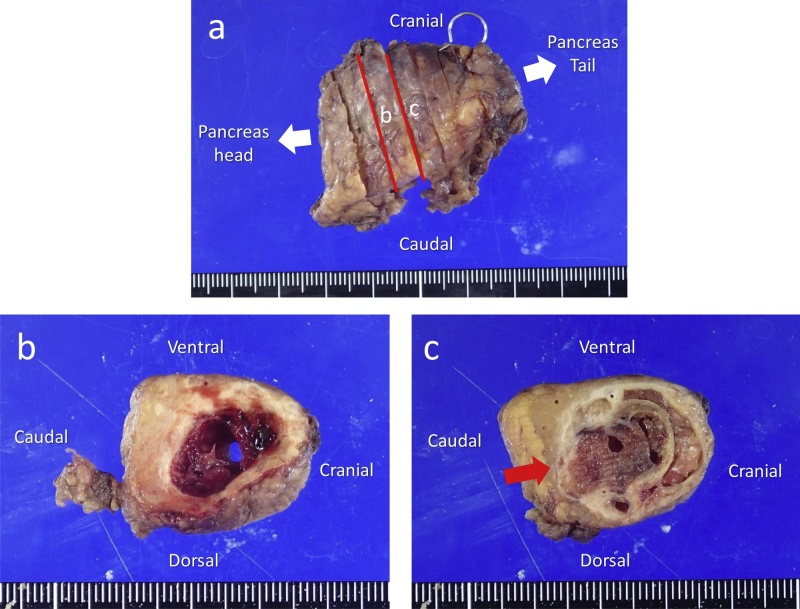
Macroscopic examination of the resected specimen **(a–c)**. The tumor is encapsulated, comprising central hemorrhage, necrosis **(b)**, and solid portions **(c)**. Main pancreatic duct (MPD) seems to be compressed from the cranial side to the caudal (a red arrow) **(c)**.

On the macroscopic examination of the resected specimen, the tumor was a solitary and encapsulated mass. The cut section revealed the solid area and the zone of hemorrhagic necrosis (Fig. 3a–c). MPD seemed to be compressed from cranial side to caudal (Fig. 3b).

**Fig. 4:**
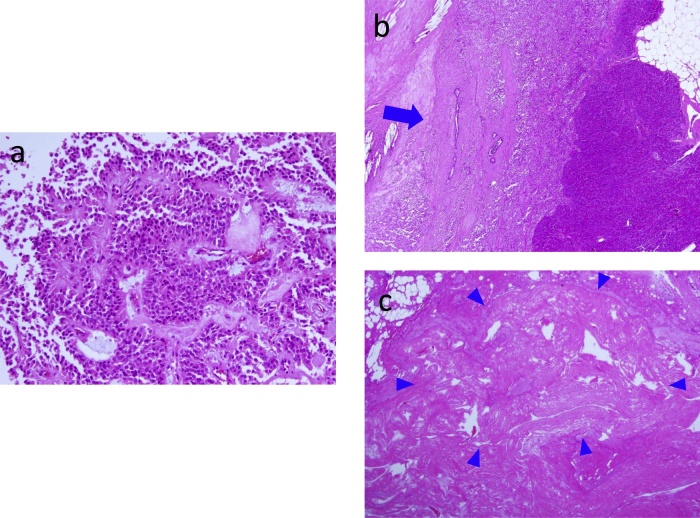
Histological examination of the surgical specimen reveals that the tumor cells have monotonous small, oval nuclei, and form the pseudo-papillary structure in a hemorrhagic background **(a)**. The tumor compresses and partly involves the main pancreatic duct (MPD) from cranial side to caudal (a blue arrow) **(b)**. In the distal atrophic parenchyma, the vestige of dilated MPD is observed (blue arrow heads) **(c)**.

**Fig. 5:**
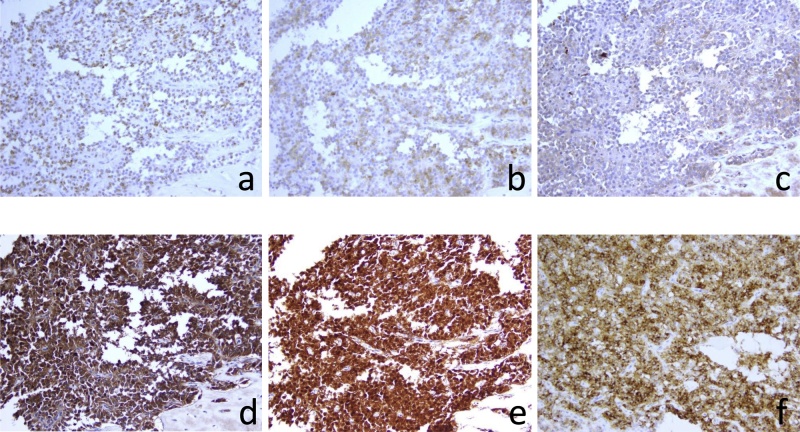
On immunohistochemical examination, the tumor cells are weakly positive for synaptophysin **(a)**, chromogranin A **(b)**, and alpha1-antitrypsin **(c)**, and positive for alpha1-antichymotrypsin **(d)**, nuclear beta-catenin **(e)**, and CD10 **(f)**.

Histologically, the tumor cells have relatively small and monotonous oval nuclei, and form the pseudo-papillary structure in a hemorrhagic background (Fig. 4a). The tumor compressed the MPD from cranial side to caudal, and partly involved MPD (Fig. 4b). In the distal atrophic parenchyma, the vestige of dilated MPD was observed (Fig. 4c). On immunohistochemical examination, the tumor cells were weakly positive for synaptophysin, chromogranin A, and alpha1-antitrypsin (Fig. 5a–c), and positive for alpha1- antichymotrypsin, nuclear beta-catenin, and CD10 (Fig. 5d–e). Consistent with the diagnosis based on EUS-FNA biopsy, we diagnosed the patient with SPN of the pancreas. Although postoperative pancreatic fistula (ISGPF Grade B) occurred from the proximal pancreas stump, she was discharged from the hospital 25 days after surgery, and has shown neither endocrine nor exocrine insufficiency.

## 3 Discussion

SPNs are considered to have low malignant potential, with an incidence of malignant transformation of 9–15% [4,5,[10], [11], [12]]. Although the optimal surgical procedure mainly depends on the tumor location, complete en-bloc resection with microscopically clear margins should be the treatment of choice with an excellent long-term prognosis [[1], [2], [3], [4], [5], [6]]. Tumor enucleation and incomplete excision should be avoided due to the tumor dissemination and the higher recurrence rate. Gao H et al. reported that positive margin might be one of the risk factors increasing the recurrence of resected SPNs [10]. Compared with other pancreatic malignancies, regional lymphatic resection is not indicated, because incidence of lymph node metastasis is extremely rare [6,[13], [14], [15]]. Considering the low-grade malignancy nature of this disease and the tumor is usually found in young female like our case, it might be reasonable to remove the tumor completely, in the meantime preserve pancreatic function by reserving the normal tissue and organs as much as possible. Intraoperative frozen section may help to ascertain the adequate resection of margins. In our case, we confirmed that no tumor invasion was observed in both proximal and distal pancreas margins during operation, and central pancreatectomy was chosen.

Accurate pre-operative histological diagnosis of SPN is very important because of the non-aggressive behavior of the tumor compared with other malignant pancreatic tumors. It has been reported that minimally invasive surgery for SPN can achieve a favorable curative effect [4,6]. Recently, EUS-FNA has become a useful diagnostic tool for patients with pancreatic tumors. The sensitivity and specificity of EUS-FNA for pancreatic neoplasms have been reported to be 91% and 94%, respectively. The overall complication rate of EUS-FNA has been reported to be low with 1% to 2% in large centers [7]. Since Nadler et al. first described a correct diagnosis of SPN based on EUS-FNA in 2002 [16], several SPN cases in which the diagnosis was made using this method have been reported. Song et al. summarized the cytopathologic features in 43 cases of SPN diagnosed based on EUS-FNA described in the English literature [17]. They reported that the cytomorphologic features observed in EUS-FNA are highly characteristic and distinct from those of other cystic or solid tumors of the pancreas.

SPNs have been reported that they rarely obstruct the MPD and cause atrophy of the distal pancreas parenchyma even if their tumor sizes are large [8]. Baek et al. reported that out of 30 cases of SPN with more than 3 cm in diameter, MPD dilatation and parenchymal atrophy were observed in only 4 (13.3%) and 2 cases (6.7%), respectively [8]. Akiyama et al. reported the case of SPN causing occlusion of the MPD and marked pancreatic atrophy distal to the tumor [18]. They hypothesized that when a tumor originated posterior to the MPD, it displaces the pancreas including the MPD, leading to serious damage to the pancreas. In our case, the tumor compressed and partially involved MPD from cranial side to caudal, which might have caused parenchymal damage of distal pancreas. In addition, atrophic distal pancreas was thought to lose its exocrine function with no acinar cells existing pathologically, we decided to close the distal stump and omitted the reconstruction. To the best of our knowledge, this is the first report of central pancreatectomy without anastomosis for the treatment of SPN. In our case, no dilatation of distal MPD was observed in preoperative images, and only the vestige of dilated MPD existed pathologically, which indicates that it has passed a long period of time since the tumor obstructed MPD.

## 4 Conclusions

We successfully performed central pancreatectomy without reconstruction for SPN located in the pancreas body with marked distal atrophy. It is important for surgeons to select the best procedure that strikes a balance between resection of the tumor and reduction of operative invasion.

## Conflicts of interest

Nothing to declare.

## Sources of funding

Nothing to declare.

## Ethical approval

This study is exempt from ethical approval in our institution.

## Consent

Written informed consent was obtained from the patient for publication of this case report and accompanying images. A copy of the written consent is available for review by the Editor-in-Chief of this journal on request.

## Author contribution

MT participated in the care of the patient including the operation and wrote the initial draft of the manuscript. TW prepared the pathological findings and revised the manuscript. SM performed in the endoscopic examination and revised the manuscript. HI, HM, and TK participated in the surgery and revised the manuscript. KK, SS, KI, and SF participated in the discussion and critically reviewed the manuscript.TK, MY, YO, and MI gave final approval of this paper to be published. All authors read and approved the final manuscript.

## Registration of research studies

Not applicable.

## Guarantor

Masanori Tsujie.

## Provenance and peer review

Not commissioned, externally peer-reviewed.
